# STI tests and proportion of positive tests in female sex workers attending local public health departments in Germany in 2010/11

**DOI:** 10.1186/s12889-016-3847-6

**Published:** 2016-11-21

**Authors:** Viviane Bremer, Karin Haar, Martyna Gassowski, Osamah Hamouda, Stine Nielsen

**Affiliations:** Department for Infectious Disease Epidemiology, Unit for HIV/AIDS, STI and Bloodborne Infections, Robert Koch-Institute, Berlin, Germany

**Keywords:** Sex workers, sexually transmitted diseases, HIV, Community health service

## Abstract

**Background:**

In Germany, local public health departments (LPHD) are required to offer low-threshold access to confidential counselling and testing for sexually transmitted infections (STI) for sex workers. We collected data from LPHD in Germany to estimate the number of performed STI tests and the proportion of positive STI tests among attending female sex workers (FSW) in order to formulate recommendations for improving STI testing and care for FSW in Germany.

**Methods:**

We recruited LPHD across Germany to collect aggregated data on attending FSW between January 2010 and March 2011. Baseline characteristics, the number of attending FSW, STI tests (HIV, *Chlamydia trachomatis*, *Neisseria gonorrhoea*, *syphilis* and *Trichomonas vaginalis*) and the number of positive results were provided by participating LPHD. We described the number of STI tests per FSW visit and the proportion of positive test results, including interquartile range (IQR). We tested whether baseline characteristics of LPHD were associated with the proportion of positive test results.

**Results:**

Overall, 28 LPHD from 14 of the 16 federal states reported 9284 FSW visits over the study period, with a median of 188 FSW visits (IQR 45–440) per LPHD. Overall, a median of 77.1% (IQR 60.7–88.0) of visiting FSW received a test for *Neisseria gonorrhoea*, followed by HIV (66.0%, IQR 47.9–86.8), *Chlamydia trachomatis* (65.4%, IQR 50.7–83.6) and syphilis (61.6, IQR 48.6–78.6). In total, 22,914 STI tests were performed. The proportion of positive tests was 3.1% (IQR 1.3–4.8), with the highest proportion of positive tests for *Chlamydia trachomatis* (6.8%, IQR 2.5–10.4), followed by *Neisseria gonorrhoea* (3.2%, IQR 0.0–5.3), *Trichomonas vaginalis* (3.0%, IQR 0.0–15.4), *syphilis* (1.1%, IQR 0.0–1.3) and HIV (0.2%, IQR 0.0–0.4). The proportion of positive tests varied between 0 and 13.9% between LPHD, with a higher variation of proportion of positive tests in LPHD with a smaller number of reported STI tests.

**Conclusions:**

Participating LPHD varied in terms of performed STI tests and FSW visits. The proportion of positive STI tests was low, but varied between LPHD. This variation likely reflects different testing strategies. Existing testing guidelines should be used by all LPHD to ensure high quality care for FSW.

**Electronic supplementary material:**

The online version of this article (doi:10.1186/s12889-016-3847-6) contains supplementary material, which is available to authorized users.

## Background

### Situation of FSW in Germany

Sex work is legal and not considered immoral in Germany since the Act Regulating the Legal Situation of Prostitutes (Prostitution Act) came into force in January 2002. According to the Prostitution Act, sex workers have the right to access health insurance and obtain social benefits such as unemployment benefits and pension. In contrast, sex workers who are self-employed without a previous health insurance in Germany or in their home country (if they are EU citizens) have difficulties to obtain health insurance and access regular health care in Germany. Sex workers without a residence permit have no access to health care insurance or to health care, unless they cover the costs themselves.

Apart from the regular insurance-based health system, local public health authorities (LPHD) are required by law to run STI clinics offering confidential counselling and testing for sexually transmitted infections (STI) for persons with a higher risk for STI. Hence, this offer also addresses female sex workers (FSW). In addition, all LPHD are required to offer anonymous and free HIV testing. STI tests are usually free of charge. There are no national guidelines regarding which STI tests should be offered by LPHD. FSW can choose to attend LPHD, or regular insurance-based health services to get tested for STI. FSW may prefer LPHD since they can guarantee anonymity and provide additional services, such as counselling on sex work, general medical care or free condoms.

### Prevalence of STI in FSW

Although FSW are often considered to be at a higher risk for STI [1–3], this may not always be the case in practise. A cross-sectional study among migrant FSW in Catalonia showed that the prevalence of *Chlamydia trachomatis* (CT) and *Neisseria gonorrhoeae* (NG) were similar to sexually active young people [4]. A higher prevalence of STI was observed in other studies among FSW with migration background and working on the street [5–8].

In Germany, only few data are available on STI. After the introduction of the Infection Protection Act in 2001, only syphilis and HIV infections remained notifiable [9, 10]. The Infection Protection Act also put an end to biweekly to monthly compulsory gynaecological examinations and tests for syphilis and NG in FSW. The change has led to fears that STI would increase in FSW. Indeed, a national increase in the number of reported syphilis infections has been observed between 2001 and 2004 and again from 2010, but this has mostly been attributed to increased transmission among men who have sex with men [11, 12].

To collect data on STI, we were running a sentinel system in the years 2003–2009 [13]. During this period, selected LPHD, STI clinics in hospitals and private practitioners submitted regular reports on patients with STI on a voluntary basis. In addition, patients with a confirmed STI-diagnosis were asked to fill in a corresponding form on migration background, social status and sexual behaviour. Overall, 4056 individual patient forms from women were returned, of which 66% were FSW. Most of the FSW were tested in LPHD [14]. While the sentinel system allowed collecting information on FSW with STI, it did not allow an estimation of the proportion of positive tests among these women, as no information on the total number of STI tests performed among FSW was available.

Subsequent to this sentinel surveillance study, we set up a cross-sectional study of selected LPHD in Germany to estimate the number of performed STI tests and the proportion of positive STI tests among FSW attending LPHD.

In this manuscript we aim to describe STI testing patterns and the proportion of positive tests and relate them to the LPHD structure in Germany.

## Methods

### Setting and study population

In 2010, Germany had a total of 414 LPHD. We invited the 62 LPHD who had taken part in the STD sentinel network to participate in the study. We asked participating LPHD to collect aggregated data on FSW attending LPHD. Between January 2010 and March 2011, we collected testing and positivity rates of different STI among FSW in participating LPHD. All study sites had previously participated in the STI-sentinel-surveillance study and were known to see many FSW [13].

As in the STI sentinel system, a FSW was defined as a woman who had at least once exchanged sex for money, drugs or goods within the past six months.

### Data collection

#### Basic survey

A basic questionnaire was sent to the LPHD participating in the STI-Sentinel in December 2009 to obtain information on their facilities, size of the facilities, number and characteristics of their attendees and tests offered. A total of 44 LPHD participated in this basic survey. Following the basic survey, a questionnaire and sampling strategy was developed in collaboration with LPHD.

#### Monthly questionnaire

The number of attending FSW, STI tests performed on FSW and number of STI diagnosis were provided in an aggregated format using a monthly or quarterly submitted form, filled out by the LPHD physicians. Decisions about who to test, which infections to test for and what types of tests to perform, was all subject to the standard testing procedures of the LPHD. The questionnaire was used to collect information on the number of tests and diagnoses of the following infections: HIV, CT, NG, *Trichomonas vaginalis* (TV), syphilis, ureaplasma, mycoplasma, hepatitis A, B, and C, and bacterial vaginosis.

#### Case definitions

The staff of the participating LPHD was asked to use case definitions for HIV, CT, NG, TV, syphilis. The case definitions can be found in the Additional file 1.

### Data analysis

We described the characteristics of participating LPHD, including the number of STI offered by LPHD (HIV, CT, NG, syphilis, TV, hepatitis A, B and C). We categorized the LPHD according to their geographic location (West-Germany (including Berlin) vs. East Germany), the covered population size (<250,000, 250,000– < 500,000, ≥500,000 residents), the level of urbanisation (urban, solely covering the population of one city vs. rural, covering the population of a county) and the monthly number of FSW and non-FSW attendees (1–25, 26–50, 51–100, >100 attendees). We performed a descriptive analysis of the number of FSW attending LPHD, the number of STI tests performed per FSW visit and the proportion of positive test results, including median and interquartile range (IQR). We excluded data on ureaplasma and mycoplasma from the analysis, since tests were only offered by four and three LPHD, respectively. For the analysis of the proportion of positive tests, we also excluded data on hepatitis A, B and C, since the aggregated data from the monthly questionnaire did not allow a differentiation between an acute infection, chronic infection or immunity.

For HIV, CT, NG, syphilis and TV, we performed a Poisson regression to test whether the proportion of positive tests differed by the size of the population covered by the participating LPHD (≤500,000 population versus >500,000), the monthly number of FSW attending the LPHD, the median proportion of FSW among all attendees or the number of STI tests performed by LPHD (≤1500 versus > 1500 tests). We used Stata 14.1®.

### Data protection issues

The data analysis was based on anonymous aggregated data provided by the LPHD. Data was entered and validated by three persons. The database was only accessible to the authors.

## Results

### Description of study sites

**Fig. 1:**
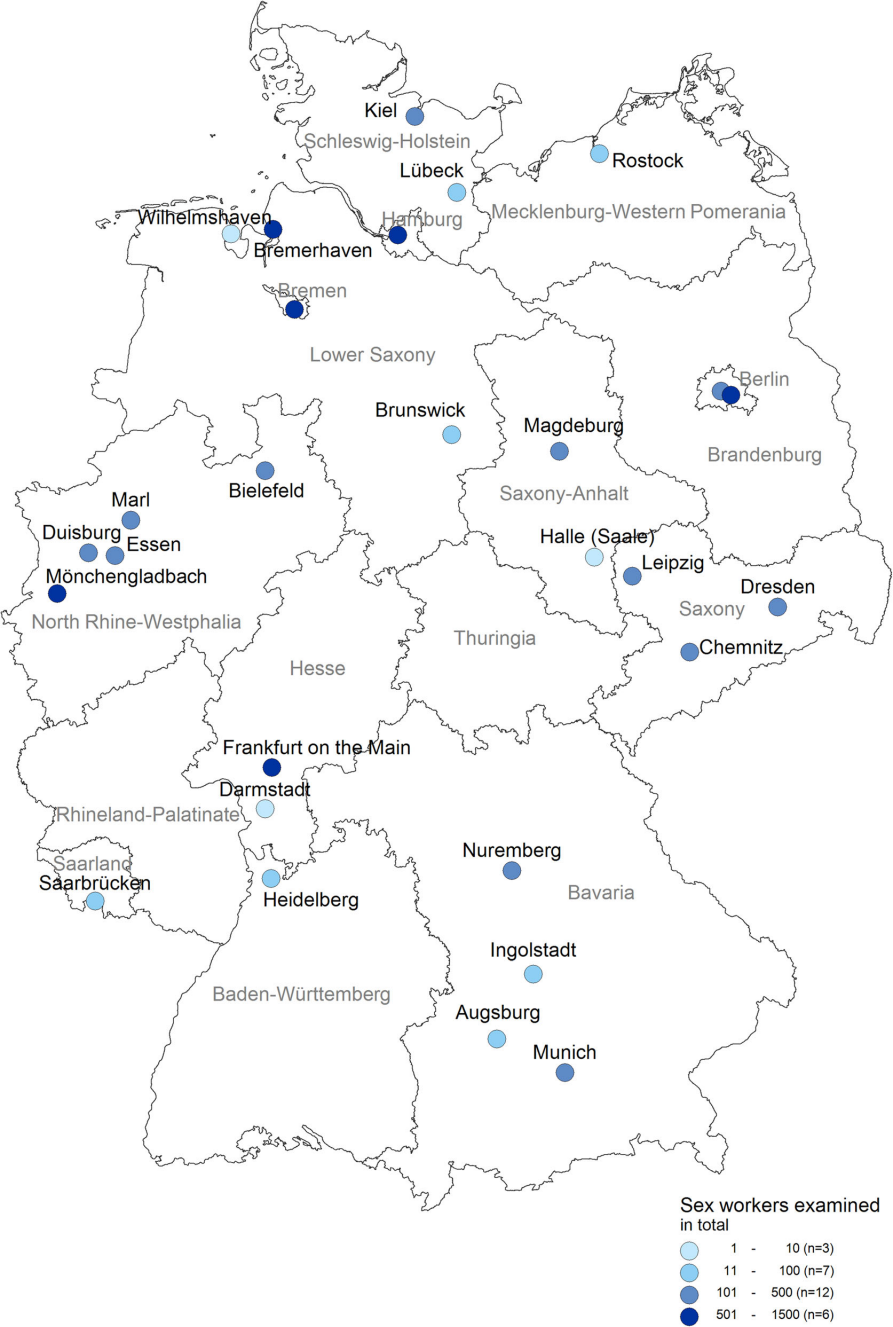
Geographical distribution of LPHD participating in the study, Germany 2010. The colour of LPHD indicates the number of included female sex workers. Source: Robert Koch-Institute

Overall, 29 LPHD from 62 LPHD that participated in the STD sentinel network (47%) agreed to participate in the study. Participating LPHD were located in 14 of the 16 German federal states. One LPHD did not see any FSW during the study period and was therefore excluded from the analysis. Of the remaining 28 LPHD, 26 (93%) were covering urban districts and two (7%) were covering rural districts. Of the LPHD covering urban districts, ten (36%) were located in cities with a population above 500,000 residents. Two (7%) LPHD were located in two different inner-city districts of Berlin. The geographical distribution of the participating LPHD and the number of FSW included is shown in Fig. 1. Twelve (43%) LPHD reported seeing 50 or less attendees per month, while fifteen (54%) saw more than 50 attendees (Table 1). Ten (36%) participating LPHD estimated that more than half of their attendees were FSW. The median proportion of FSW among the attendees seen was 20% (range 1–99%).

**Table 1:** Characteristics of participating LPHD in Germany. *N* = 28

LPHD characteristics		West incl. Berlin	East	Total
Population size covered	<250,000	10	4	14
	250,000- < 500,000	4	0	4
	≥500,000	8	2	10
Monthly number of attendees	1–25	8	1	9
	26–50	1	2	3
	51–100	5	2	7
	>100	7	1	8
	Missing	1	0	1
Proportion of FSW among attendees	1–25%	10	4	14
	26–50%	3	0	3
	51–75%	2	0	2
	76–100%	6	2	8
	Missing	1	0	1
Number of STI tests offered^a^	Only HIV-test	1	0	1
	HIV plus 2–6 STI tests	5	1	6
	HIV plus 7 STI tests	16	5	21

^a^STI tests: HIV, syphilis, *Chlamydia trachomatis*, *Neisseria gonorrhoea*, *Trichomonas vaginalis*, hepatitis A, hepatitis B, hepatitis C

All (100%) participating LPHD reported to offer HIV tests on a routine basis. Six (21%) offered three to six additional STI tests and 21 (81%) LPHD offered seven different STI tests. CT and syphilis tests were offered by 27 (96%) LPHD each, 26 (93%) LPHD offered testing for NG and 18 (64%) LPHD offered testing for TV. Tests for hepatitis A were offered by 20 (71%) LPHD, hepatitis B and C tests were each offered by 24 (86%) LPHD.

### FSW visits

Participating LPHD reported a total of 9284 visits of FSW throughout the 15-month-long study period. This corresponds to a median of 188 FSW visits (IQR 45–440) per LPHD. Overall, 60.7% of all FSW visits were recorded in LPHD covering a population of more than 500,000 residents. The highest number of FSW visits during the study period was reported by the LPHD in Frankfurt on the Main (*n* = 1344), followed by Hamburg (*n* = 1337). Three LPHD reported less than 10 FSW visits over the study period. The LPHD performed a median of 2.9 STI and HIV tests per FSW visit (IQR 2.3–3.4).

### STI tests performed

In total, 22,914 tests for HIV, CT, NG, syphilis and TV were performed on FSW within the study period. Visiting FSW were most frequently tested for NG (*n* = 6005) and CT (*n* = 5353). The highest number of STI tests in FSW were reported by the LPHD in Frankfurt on the Main (*n* = 3851) and Hamburg (*n* = 2978). Overall, a median of 77.1% (IQR 60.7–88.0) of visiting FSW received a NG test, followed by HIV (66.0%, IQR 47.9–86.8), CT (65.4%, IQR 50.7–83.6) and syphilis (61.6%, IQR 48.6–78.6). TV was tested for in 45.2% (IQR 14.2–71.6) of the FSW visits (Table 2).

**Table 2:** STI tests per LPHD, FSW visit, and positive STI tests

Tests	Number of LPHD offering test (%)	Number of performed tests	Median number tests per LPHD^a^	Median proportion of tests per FSW visit in % (IQR)^a^	Number of FSW visits with positive test	Proportion of positive tests in % (IQR)^a^
HIV	28 (100)	3882	86.5	66.0 (47.9–86.8)	8	0.2 (0.0–0.4)
*Chlamydia trachomatis*	27 (96)	5353	94.5	65.4 (50.7–83.6)	366	6.8 (2.5–10.4)
*Neisseria gonorrhoea*	26 (93)	6005	143.5	77.1 (60.7–88.0)	195	3.2 (0.0–5.3)
Syphilis	27 (96)	4474	135.0	61.6 (48.6–78.6)	50	1.1 (0.0–1.3)
*Trichomonas vaginalis*	18 (64)	3200	67.0	45.2 (14.2–71.6)	97	3.0 (0.0–15.4)

*IQR* interquartile range

^a^includes only those LPHD offering the respective test

### Positive tests

**Fig. 2:**
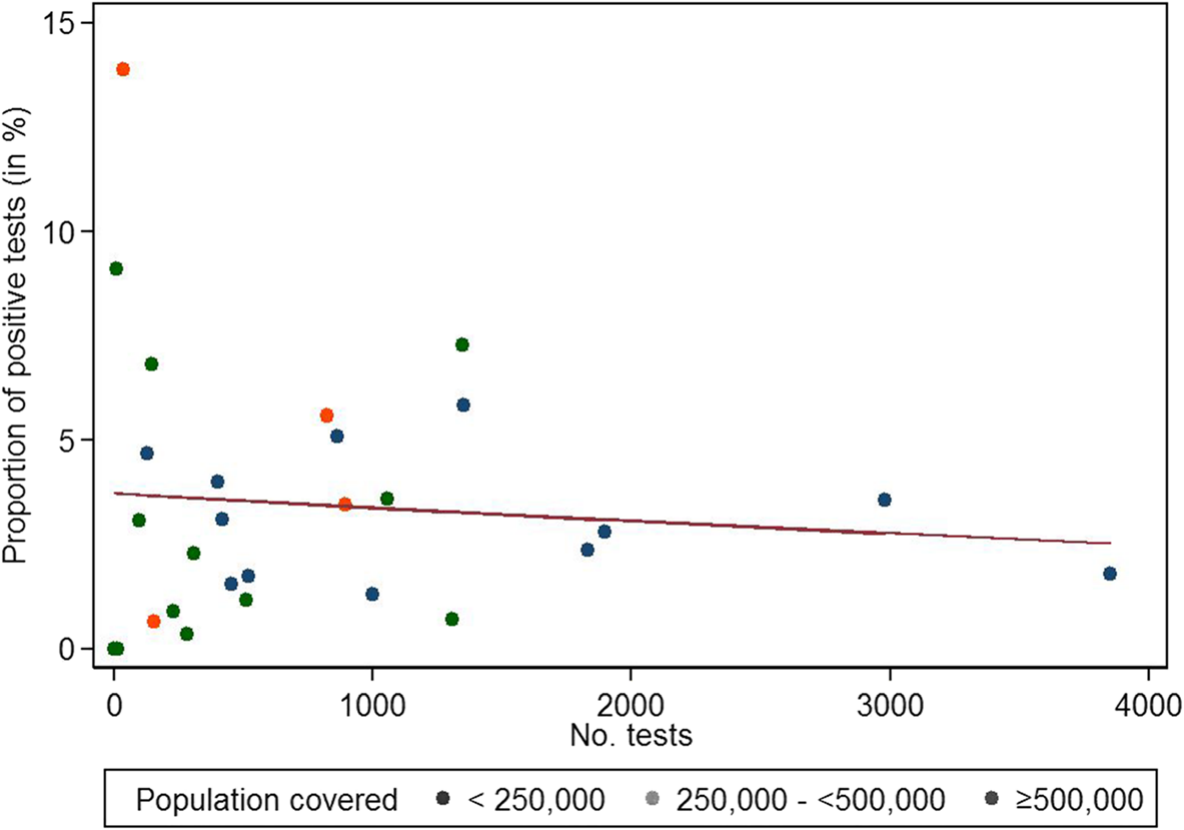
Proportion of positive STI tests per LPHD by number of performed STI tests and covered population size, including trend line, Germany 2010–11

Overall, 716 (3.1%, IQR 1.3–4.8) of all STI tests were positive. The largest number of positive test results was from Hamburg (106; 14.8%), followed by Bremerhaven (98; 13.7%). The proportion of positive STI tests was the highest for CT (6.8%, IQR 2.5–10.4), followed by NG (3.2%, IQR 0.0–5.3), TV (3.0%, IQR 0.0–15.4) and syphilis (1.1%, IQR 0.0–1.3). The lowest proportion of positive tests was observed for HIV with 0.2% (IQR 0.0–0.4) (Table 2). The proportion of positive STI tests per LPHD varied between 0.0 and 13.9%. The proportion of positive CT tests ranged between 0.0–50.0%. For NG, this proportion ranged from 0.0 to 9.5%, for syphilis between 0.0 and 12.5% and TV 0.0–67.6%. The proportion of positive STI tests varied between 0.0 and 13.9% among LPHD with up to 1500 STI tests and 1.8 and 3.6% among LPHD with more than 1500 STI tests during the study period, but this association was not significant (*p* = 0.340). The proportion of positive tests did not differ by population covered by LPHD (*p* = 0.462). The proportion of positive STI tests decreased slightly, but not significantly with a higher number of performed STI tests (*p* = 0.404) (Fig. 2).

**Fig. 3:**
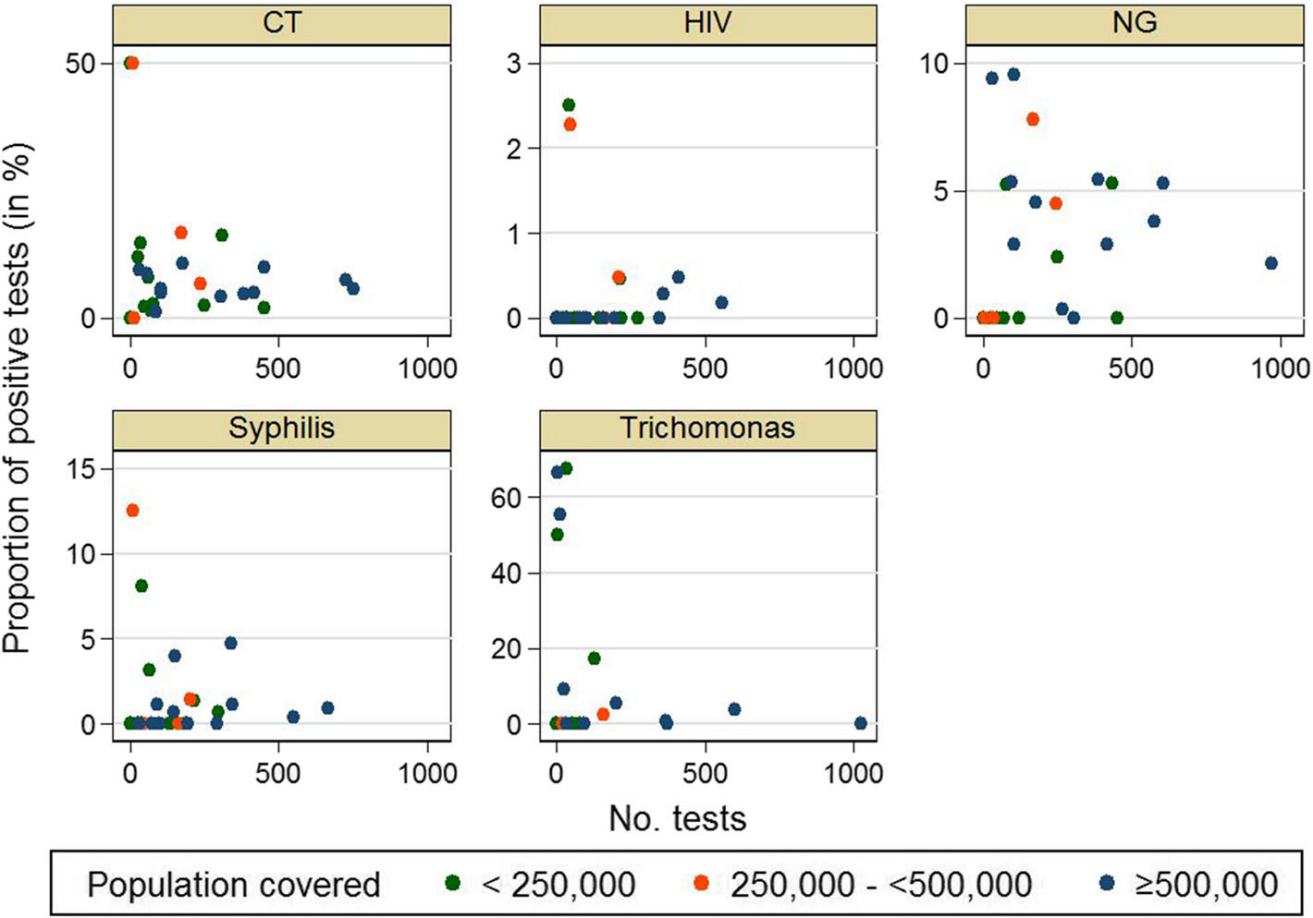
Proportion of positive CT, NG, HIV, syphilis and TV tests according to covered population size of the participating LPHD in Germany 2010–11

The proportion of positive tests also varied by STI tested (Fig. 3). The highest variation in the positive proportion could be observed in NG and TV, the lowest in HIV. The Poisson regression showed that the proportion of positive tests for each of the different STI did not differ by the size of the population covered by the participating LPHD, the monthly number of FSW attending the LPHD, the median proportion of FSW among all attendees or the number of STI tests performed by LPHD.

## Discussion

Participating LPHD show a large variation in terms of STI tests offered and the number of FSW visits for counselling and testing. The overall proportion of positive STI tests was relatively low. However, the proportion of positive tests highly varied between the LPHD, with a higher range of the proportion of positive STI tests among LPHD with smaller numbers of performed STI tests.

### Variation of STI tests offered by LPHD

The study revealed different baseline characteristics among the participating LPHD such as the number of attendees, the proportion of FSW among all attendees and the number and type of STI tests offered. These differences have also been observed in a survey among 250 German LPHD from 2012 [15]. In this survey, fifty-six percent of the participating LPHD reported testing for syphilis, and 27 and 28% offered tests for CT and NG, respectively. Thus, the proportion of LPHD in Germany offering several STI tests seems even lower than what we found in our study.

The observed differences between the number of attending FSW as well as the number of STI tests among LPHD may be partly explained by the size of the population covered and the number of FSW working in the covered district. However, the number of FSW attending the LPHD may also depend on what is offered by the site. For example, if a LPDH has limited staff resources and reduced consultation hours, FSW will have more difficulties to attend the LPHD. Unfortunately, we did not assess staff resources and consultation hours; thus the impact of accessibility should be subject of future studies. Acceptance of healthcare services and hence frequent utilisation of LPHD might be enhanced by embedding STI tests into general gynaecological care and offering services tailored to FSW as it has been observed in the UK [16].

In Germany, LPHD are run and financed by city or district authorities. We observed a larger range of the proportion of positive tests among LPHD with small numbers of performed STI tests compared to LPHD with high testing numbers. This observation points towards different testing strategies. The 2012 guidelines of the International Union against Sexually Transmitted Infections (IUSTI) recommend that all patients attending facilities dealing with STI should be screened for CT, NG, syphilis, and HIV. Additional STI tests such as TV, LGV, hepatitis B or C should be offered according to sexual history and examination findings [17]. Although LPHD should offer tests for the same STI throughout the country, the low median number of STI test per FSW visit indicates that only few LPHD in Germany seem to follow the current IUSTI guidelines. Some LPHD may only offer STI tests in the presence of symptoms such as vaginal discharge while others may be applying a more screening-based approach. International clinical guidelines are often unknown in Germany and non-adherence to these guidelines is not sanctioned. Therefore, the decisions on what STI tests should be offered to the patient seems to be primarily guided by the local financial situation and political support of the LPHD.

### Low proportion of positive STI tests

Overall, we observed low proportions of positive STI tests, particularly for HIV (0.2%). Higher HIV prevalence was found in a study of indoor-working FSW in the UK (1.1%) and in a convenience sample of FSW in Catalonia (1.5%) [1, 18]. On the other hand, newer data from Spanish HIV/STI clinics and data from STI clinics in the Netherlands show 0.8 and 0.1%, of FSW infected with HIV, respectively [19, 20]. These differences between studies may reflect a real difference in HIV incidence and prevalence, as the reported incidence of HIV infections in females in 2011 was higher in the UK and Spain than in the Netherlands and Germany [21]. Generally, HIV prevalence appears to be low in Europe among FSW who do not inject drugs [22]. Alternatively, the observed proportion of positive HIV tests may have been low since we only assessed test results from FSW attending LPHD. FSW who do not have any access to health care may have a higher HIV prevalence as suggested by findings in Peru [23]. In the Netherlands, Verscheijden et al. observed a lower STI prevalence during outreach activities than at STI clinics, but this may explained by the fact that outreach activities mainly targeted FSW working in legal establishments [19, 23].

The highest proportion of positive tests was seen for CT (6.8%). Only few STI prevalence studies among FSW in high income countries have been published. The proportion of positive CT (6.8%) and NG tests (3.2%) in our study was higher than in a Spanish study among 400 FSW, with a proportion of 5.5 and 0.6%, respectively. Platt et al. observed a prevalence of 4.3 and 2.2% in the UK [1, 24]. Data from STI clinics in the Netherland suggest that the proportion of positive CT tests was lower in FSW (7.2% vs. 11.5%) whereas the proportion of positive NG (2.5% vs. 1.2%) and syphilis (0.2% vs. 0.02%) were higher, compared to women not reporting sex work [25]. Until recently, only few data existed on CT infections in Germany. But through the German Chlamydia Laboratory Sentinel system, data is now collected from selected laboratories on performed CT tests, especially among pregnant women and women <25 years (who in Germany are entitled to CT screening). The proportion of positive CT tests in all women included in the Laboratory Sentinel system was 3.9% and therefore lower than the proportion found in FSW in our study. However, the proportion of positive CT tests in 15–19 and 20–24 year-old women was similarly high with 6.8 and 6.0%, respectively [26]. The risk of acquiring CT may be comparable between young women and FSW and it is difficult to conclude whether FSW have a substantially higher CT prevalence than the general female population. As for NG and syphilis, the proportion of positive tests in FSW cannot be compared to the general population as no similar data are available for Germany.

### Limitations

This study has some limitations. LPHD volunteering to participate in this study may have been more likely to offer more STI tests than non-participating LPHD. Therefore, the baseline characteristics of participating LPHD may not be representative for all German LPHD.

As we only received aggregated data for our analysis of all FSW attending LPHD, it was not possible to differentiate between FSW visiting once or several times during the study period. FSW attending LPHD on a regular base may have had a lower probability of being tested positive. This would have led to an underestimation of the proportion of positive STI tests in this study. It is also possible that not all FSW disclosed their occupation, in which case the number of attending FSW would be underestimated.

The chance of discovering an STI was higher in FSW receiving all recommended tests (HIV, CT, NG, syphilis), and potentially lower in FSW receiving only a few tests, resulting in a possible underestimation of the number of STI in the latter group. In addition, as all LPHD offer anonymous HIV tests, they were not able to document the exact number of HIV tests among FSW. Thus, the proportion of FSW tested for HIV may have been underestimated.

FSW attending LPHD may not be representative of all FSW. While FSW with regular health insurance may prefer to attend regular health services, migrant FSW, especially those without German language skills, may be less likely to attend any healthcare facilities.

## Conclusions

In conclusion, STI testing offered by LPHD should be harmonised in Germany. Ideally, the decision of offering an STI test to a FSW should be guided by clinical guidelines. In addition, LPHD staff should take the individual sexual history and clinical symptoms of the FSW into account to guide additional testing. Therefore, it is important to establish commonly agreed testing standards in LPHD. Especially CT, TV and hepatitis B and C tests should be offered on a larger scale. Recently, the German STI Society issued guidelines for STI counselling, diagnostics and treatment [27]. These recommendations are adapted to the German context and should be used as a gold standard in daily practise.

## Additional file

Additional file 1: Case definitions used in the study. (DOCX 33 kb)

## Abbreviations

CI: Confidence intervals
CT: *Chlamydia trachomatis*
FSW: Female sex workers
HIV: Human immunodeficiency virus
LPHD: Local public health departments
NAAT: Nucleic acid amplification test
NG: *Neisseria gonorrhoeae*
STI: Sexually transmitted infections
TV: *Trichomonas vaginalis*
