# Supplementary material for: STI tests and proportion of positive tests in female sex workers attending local public health departments in Germany in 2010/11
Source: BMC Public Health. 2016 Nov 21;16:1175. doi: 10.1186/s12889-016-3847-6 (PMC5117614; doi:10.1186/s12889-016-3847-6)
Supplement: Additional file 1: — Case definitions used in the study. (DOCX 33 kb) [file 12889_2016_3847_MOESM1_ESM.docx]

**Additional file 1: Case definitions used in the study**

1. HIV - laboratory diagnosis with at least one of the following tests: virus culture, antibody test (confirmed by Western Blot or immunofluorescence test), HIV point-of-care test
2. CT - laboratory diagnosis with at least one of the following tests: culture, fluorescent antigen test, ELISA or nucleic acid amplification test (NAAT) from cervical, rectal, pharyngeal or urinary samples
3. NG - laboratory diagnosis with at least one of the following tests: isolation of gram-negative oxidase-positive diplococci (*Neisseria gonorrhoeae*) from urethral, rectal, cervical, vaginal or pharyngeal samples; NAAT
4. Trichomoniasis - microscopic evidence of *Trichomonas vaginalis* in wet preparations of genital secretions, urethral swab or urinary sediment after centrifugation
5. Syphilis

- clinical picture consistent with primary, secondary or tertiary stage and laboratory diagnosis with at least one of the following tests:
  - dark-field microscopy or fluorescence microscopy
  - Detection of *Treponema pallidum* antibodies (eg: TPHA, TPPA or EIA) AND additionally detection of either Tp-IgM antibodies (eg: IgM ELISA or immunoblot or 19S-IgM-FTA-abs) OR cardiolipin non-Tp antibodies (e.g. KBR>8, VDRL>4).
- early latent syphilis (≤ 1 year): a history of symptoms compatible with those of the earlier stages of syphilis and a documented seroconversion or 4-fold or higher increase in VDRL within the previous 12 months
- late latent syphilis (> 1 year): no symptoms and a documented seroconversion or 4-fold or higher increase in VDRL
- neurosyphilis: clinical picture consistent with neurosyphilis and laboratory diagnosis as described for primary stage and an ITPA index >2
- cardiovascular syphilis: clinical picture consistent with cardiovascular syphilis and laboratory diagnosis as described for primary stage
